# Supplementary material for: Left ventricular mass normalization for body size in children based on an allometrically adjusted ratio is as accurate as normalization based on the centile curves method
Source: PLoS One. 2019 Nov 21;14(11):e0225287. doi: 10.1371/journal.pone.0225287 (PMC6872180; doi:10.1371/journal.pone.0225287)
Supplement: S1 Table — (DOCX) [file pone.0225287.s005.docx]

**S1 Table. The equation to calculate Lin’s concordance correlation coefficient and equations to calculate the intermediate factors.**

| Coefficient / Factor | Equation |
| --- | --- |
| Concordance correlation coefficient (CCC, *r_c_*) | $r_{c}=\frac{2rs_{x}s_{y}}{s_{x}^{2}+s_{y}^{2}+\left( \overline{x}-\overline{y} \right)^{2}}$ |
|  | $r_{c}=rc_{b}$ |
| Bias correction factor (*c_b_*) | $c_{b}=\frac{2}{\left( \frac{v+1}{v+u^{2}} \right)}$ |
| Scale shift (*v*) | $v=\frac{s_{x}}{s_{y}}$ |
| Location shift (*u*) | $u=\frac{\overline{x}-\overline{y}}{\sqrt{s_{x}s_{y}}}$ |

Here *r* is the Pearson correlation coefficient for a relationship between x and y. The Pearson correlation coefficient is a measure of precision. *s_x_, s_y_*  are the standard deviations of the compared *x* and *y* data sets, respectively; $\overline{x},\overline{y}$ are the means of the compared *x* and *y* data sets, respectively. *c_b_* is the bias correction factor - a measure of accuracy. Reference: Lin LI. A concordance correlation coefficient to evaluate reproducibility. Biometrics. 1989 Mar; 45(1): 255-68. PMID: 2720055.
