# Supplementary material for: Left ventricular mass normalization for body size in children based on an allometrically adjusted ratio is as accurate as normalization based on the centile curves method
Source: PLoS One. 2019 Nov 21;14(11):e0225287. doi: 10.1371/journal.pone.0225287 (PMC6872180; doi:10.1371/journal.pone.0225287)
Supplement: S1 Text — (DOCX) [file pone.0225287.s006.docx]

**Examples of LVM z-score calculations**

Computation of LVM z-score for an individual child, from the L, M, and S values corresponding to the child’s height.

**Child 1**: 12-year-old girl, 167 cm in height, with actual LVM = 131.2040 g (observation number 154 in the S3 Dataset). The L, M, and S values corresponding to her height are 0.1892, 119.8197, and 0.1503, respectively (see: S1 Dataset). The calculations are made according to equation (1) as follows:

| (1) | $z\text{-}score=\frac{\left[ \left( \frac{actual LVM}{M} \right)^{L}-1 \right]}{L\times S}$ |
| --- | --- |
| (2) | $z\text{-}score=\frac{\left[ \left( \frac{131.2040}{119.8197} \right)^{0.1892}-1 \right]}{0.1892\times0.1503}$ |
| (3) | $z\text{-}score=0.6092$ |

**Child 2**: 16-year-old boy, 185 cm in height, with actual LVM = 234.8180 g (observation number 185 in the S3 Dataset). The L, M, and S values corresponding to his height are 0.6942, 186.8259, and 0.1983, respectively (see: S1 Dataset). The calculations are made according to equation (1) as follows:

| (1) | $z\text{-}score=\frac{\left[ \left( \frac{actual LVM}{M} \right)^{L}-1 \right]}{L\times S}$ |
| --- | --- |
| (4) | $z\text{-}score=\frac{\left[ \left( \frac{234.8180}{186.8259} \right)^{0,6942}-1 \right]}{0.6942\times0.1983}$ |
| (5) | $z\text{-}score=1.2491$ |

Computation of LVM z-score for an individual child, from normative data, produced based on the LVM-for-height ratio adjusted with specific allometric exponents, expressed as a mean and standard deviation.

**Child 1**: 12-year-old girl, 167 cm (1.67 m) in height, with actual LVM = 131.2040 g (observation number 154 in the S3 Dataset). The specific allometric exponent for girls is 2.5848, and the mean allometrically adjusted LVM-for-height ratio and its standard deviation (normative data) are 32.0467 and 5.1431, respectively (see Table 3 in the article). The calculations are made according to equation (6) as follows:

| (6) | $z\text{-}score=\frac{\left( \frac{actual LVM}{{height}^{b}} \right)-\left( {mean}_{normative data}\frac{LVM}{{height}^{b}} \right)}{{standard deviation}_{normative data}}$ |
| --- | --- |
| (7) | $z\text{-}score=\frac{\left( \frac{131.2040}{{1.67}^{2.5848}} \right)-\left( 32.0467 \right)}{5.1431}$ |
| (8) | $z\text{-}score=0.5462$ |

**Child 2**: 16-year-old boy, 185 cm (1.85 m) in height, with actual LVM = 234.8180 g (observation number 185 in the S3 Dataset). The specific allometric exponent for boys is 2.8118, and the mean allometrically adjusted LVM-for-height ratio and its standard deviation (normative data) are 32.5524 and 6.1043, respectively (see Table 3 in the article). The calculations are made according to equation (6) as follows:

| (6) | $z\text{-}score=\frac{\left( \frac{actual LVM}{{height}^{b}} \right)-\left( {mean}_{normative data}\frac{LVM}{{height}^{b}} \right)}{{standard deviation}_{normative data}}$ |
| --- | --- |
| (9) | $z\text{-}score=\frac{\left( \frac{234.8180}{{1.85}^{2.8118}} \right)-\left( 32.5524 \right)}{6.1043}$ |
| (10) | $z\text{-}score=1.4885$ |

For the computation of LVM z-score for an individual child, from normative data produced based on the LVM-for-height ratio adjusted with the allometric exponent of 2.7 (a universal exponent), the same equation (6) and procedure as for the specific exponent are used. However, for both, girls and boys, the exponent of 2.7 is used instead of the specific one (b). Respective, sex-specific normative data, expressed as a mean and standard deviation, are presented in Table 3 of the article.
